# Sequence diversity within the HA-1 gene as detected by melting temperature assay without oligonucleotide probes

**DOI:** 10.1186/1471-2350-6-36

**Published:** 2005-10-04

**Authors:** Claudio Graziano, Massimo Giorgi, Cecilia Malentacchi, Pier Luigi Mattiuz, Berardino Porfirio

**Affiliations:** 1 Human Genetics Unit, Department of Clinical Physiopathology, University of Florence, Viale Gaetano Pieraccini 6, 50139 Firenze, Italy; 2 Current address: U.O. e Cattedra di Genetica Medica, Policlinico S. Orsola-Malpighi, Via Massarenti 9, 40138 Bologna, Italy

## Abstract

**Background:**

The minor histocompatibility antigens (mHags) are self-peptides derived from common cellular proteins and presented by MHC class I and II molecules. Disparities in mHags are a potential risk for the development of graft-versus-host disease (GvHD) in the recipients of bone marrow from HLA-identical donors. Two alleles have been identified in the mHag *HA-1*. The correlation between mismatches of the mHag HA-1 and GvHD has been suggested and methods to facilitate large-scale testing were afterwards developed.

**Methods:**

We used sequence specific primer (SSP) PCR and direct sequencing to detect *HA-1 *gene polymorphisms in a sample of 131 unrelated Italian subjects. We then set up a novel melting temperature (Tm) assay that may help identification of *HA-1 *alleles without oligonucleotide probes.

**Results:**

We report the frequencies of *HA-1 *alleles in the Italian population and the presence of an intronic 5 base-pair deletion associated with the immunogeneic allele HA-1^H^. We also detected novel variable sites with respect to the consensus sequence of *HA-1 *locus. Even though recombination/gene conversion events are documented, there is considerable linkage disequilibrium in the data. The gametic associations between HA-1^R/H ^alleles and the intronic 5-bp ins/del polymorphism prompted us to try the Tm analysis with SYBR^® ^Green I. We show that the addition of dimethylsulfoxide (DMSO) during the assay yields distinct patterns when amplicons from HA-1^H ^homozygotes, HA-1^R ^homozygotes, and heterozygotes are analysed.

**Conclusion:**

The possibility to use SYBR^® ^Green I to detect Tm differences between allelic variants is attractive but requires great caution. We succeeded in allele discrimination of the HA-1 locus using a relatively short (101 bp) amplicon, only in the presence of DMSO. We believe that, at least in certain assets, Tm assays may benefit by the addition of DMSO or other agents affecting DNA strand conformation and stability.

## Background

Acute graft-versus-host disease (aGvHD) is still a major cause of morbidity after allogeneic HLA-identical bone marrow transplantation, occurring in 10–60% of patients receiving matched sibling allograft, depending on prophylaxis regimen. These figures turned out to be even higher in the case of unrelated matched allograft [1]. Recent studies emphasize the involvement of mHags disparities in the development of aGvHD [2-4].

Among known autosomal mHags, only *HA-1 *has been implicated as a cause of aGvHD in humans [2]. *HA-1 *is a nonapeptide from a protein encoded by a gene termed KIAA0223 (GenBank accession no. D86976), a polymorphic gene that has two known alleles differing at positions 500 and 504 of the cDNA sequence, resulting in a single aminoacid change. The HA-1^H ^allele encodes histidine at position 3 of the peptide, is recognized by HLA-A*0201-restricted cytotoxic T cells and is only expressed by cells of haematopoietic origin [5]. Its allelic counterpart, HA-1^R^, encodes arginine at position 3. HLA-A*0201 molecules have low affinity for the HA-1^R ^peptide and the complex does not generate a detectable immune response [5]. *HA-1 *disparity can thus be defined as the presence of HA-1^H ^in the recipient but not in the donor, because in such cases T cells in the transplanted donor marrow respond to mHags in the recipient.

Four different DNA-based strategies have been described so far to perform *HA-1 *allelotyping. They rely on sequence specific primer (SSP) PCR [6], restriction fragment length polymorphism (RFLP) PCR [7], reference strand mediated conformation analysis (RSCA) [8], and allele specific fluorescence-labelled probes [9].

## Methods

### HA-1 allelotyping

**Figure 1:**
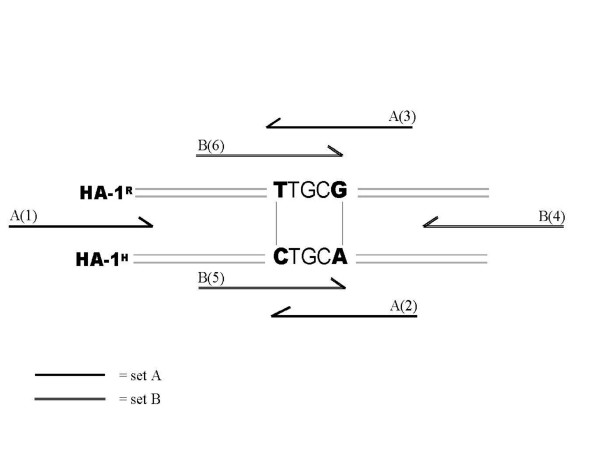
Relative positions of primer pair sets A and B designed to perform allele-specific PCR typing at the *HA-1 *locus. A(1) is the forward primer common to site-specific primers A(2) and A(3) designed with the 3'OH-end at c.500C/T. B(4) is the reverse primer common to site-specific primers B(5) and B(6) designed with the 3'OH-end at c.504A/G. Primer combinations A(1) + A(2) and B(4) + B(5) are specific for HA-1^H^. Primer combinations A(1) + A(3) and B(4) + B(6) are specific for HA-1^R^.

We performed *HA-1 *typing through allele-specific PCR as described by Wilke et al [6], with minor modifications. Two different primer sets (designed on both strands) were used: each set contained a common external primer and specific primers for allele HA-1^H ^and HA-1^R ^(Figure 1).

The two common primers were used to generate a fragment of an expected length of 486 bp containing the polymorphic sites. Relative positions of primer pair sets A and B designed to perform allele-specific PCR typing at the *HA-1 *locus are reported in figure 1. Forty ng of genomic DNA was used in 50 μL of a reaction mixture containing Applied Biosystems PCR Buffer with 1.5 mmol/L MgCl_2_, 15 pmol of each primer, 0.8 mmol/L dNTPs, and 2 units of AmpliTaq polymerase (Applied Biosystems). Cycling conditions were according to Wilke et al [6]. Amplicons were purified with the QIAquick Purification Kit (Qiagen). Sequencing was performed on the ABI PRISM 310 Genetic Analyzer according to the protocol provided by the manufacturer.

### Melting curve analysis

The primers used to amplify a 101-bp fragment were: forward, 5'-CTTCGCTGAGGGCCTTGAG-3' and reverse, 5'-CCTTGGGTCTGGCTCTGTCTT-3'. The reactions were performed in a total volume of 100 μL containing 50 μL of SYBR^® ^Green PCR Master Mix (Applied Biosystems), a 300/300 mmol/L forward/reverse primer combination and 50 ng of genomic DNA. The cycling conditions were: 94°C for 45 sec, 60°C for 45 sec and 72°C for 45 sec in 33 cycles. After PCR, DMSO at 5% or 10% was added to the reaction tubes and the total volume was divided in 4 wells of an optical reaction plate. The dissociation protocol was performed in accordance to the default thermal profile of the ABI 5700 Sequence Detection System (Applied Biosystems), which included, after a 15 sec hold at 95°C and a 20 sec hold at 60°C, a slow ramp (20 min) from 60°C to 95°C. The fluorescence was monitored in real time (every 3 sec) for each sample. The melting peaks were calculated by the ABI 5700 software as the negative derivative of fluorescence with respect to the temperature (-dF/dT *vs *T).

## Results and discussion

We report our experience with melting temperature (Tm) analysis, SSP-PCR, and direct sequencing in detecting the *HA-1 *polymorphism in a cohort of Italian subjects, which allowed us to disclose further polymorphic variations surrounding the two *HA-1 *alleles and, in particular, an intronic 5-bp deletion, strongly associated with HA-1^H ^allele.

We sampled 131 unrelated subjects from the Italian population, obtaining a genotypic distribution of 23 HA-1^H ^homozygotes, 41 HA-1^R ^homozygotes, and 67 heterozygotes. The resulting allelic frequencies were 0.43 for allele HA-1^H ^and 0.57 for allele HA-1^R^. The observed genotypes conformed to the Hardy-Weinberg law (*X*^2 ^= 0.23, not significant). Furthermore, our allele frequency estimates are associated with a standard error of 0.03 and are in line with those reported by Tseng et al [7] for North American Caucasians.

*HA-1 *typing was performed by allele-specific PCR as described by Wilke et al [6], with minor modifications (Figure 1). Occasionally, we have obtained ambiguous typing results using this approach. The same difficulties have been mentioned by Kreiter et al [9]. These findings prompted us to sequence the entire region. In comparison with the sequence retrieved from GenBank under accession number AF092537, which corresponds to the HA-1^R^, we identified an intronic 5-bp deletion in the first case examined. Therefore, we decided to analyse a random sample from our population by sequencing the *HA-1 *genomic region in a total of 36 subjects. The results are summarised in Table 1. Twenty-seven of 72 chromosomes (37.5%) showed the 5-bp deletion. Further polymorphic variation was detected at c.509+65 and at c.509+130. Four of 72 chromosomes (5.6%) showed G to A transitions at those intronic sites. Haplotypes A and B are present in the Italian population at comparable frequencies. They differ at the sites defining the HA-1^R/H ^polymorphism as well as at c.509+213 where the pentanucleotide TTTAT is missing in haplotype B. This deletion was also identified by us in subjects from Sardinia, Germany and China, indicating that it is widespread in human populations, possibly underlying its ancient origin. We deposited this sequence under GenBank accession number AF236756. In the meanwhile, the same ins/del polymorphism was reported by Aróstegui et al [8], which also described the occurrence of "del" with HA-1^H ^allele and that of "ins" with HA-1^R^. Our data showed that these gametic associations are in nearly complete linkage disequilibrium and that further variation is present within the *HA-1 *gene. Haplotype C is by far less frequent than haplotypes A and B. It is characterised by the presence of G to A transitions at c.509+65 and c.509+310 in a haplotype A context. A relatively recent gene conversion event, which may have occurred on this milieu, is the most parsimonious path we can envisage. The same ancestral haplotype A may have harboured a silent exonic transition (c.427C→T) to generate haplotype E. Whether this variation is polymorphic or merely a rare mutation remains to be verified on larger samples. We also found a single chromosome with the HA-1^R ^allele associated with the intronic deletion (haplotype D). This chromosome may well have raised from a crossing-over in a HA-1^R/H ^heterozygote.

In order to make *HA-1 *typing easier and faster, we tested the Tm approach to define *HA-1 *alleles. As reported, the ability of Tm analysis to discriminate different PCR products mainly depends on the length of the amplicon and the GC content [11,12]. Both amplification and Tm analysis are automated processes that can be performed on fluorescence-detection equipped thermal cyclers. Double-stranded DNA products are detected by the SYBR^® ^Green I nucleic acid dye in real-time during PCR. At the end of the reaction the temperature at which the transition double/single strand DNA occurs is revealed by following SYBR^® ^Green I emission decay. Different PCR products may determine specific dissociation profiles [11,12].

As a matter of fact, knowledge of the gametic associations between HA-1^R/H ^alleles and the intronic 5-bp ins/del polymorphism might allow a straightforward Tm analysis combining H/R allele specific primer and the presence of the 5-bp deletion in a single amplicon. However, the fragment would be too large to allow correct typing (data not shown). In this respect, our results are in keeping with the criticisms raised by von Ahsen et al [13] towards Tm assays based on SYBR^® ^Green I melting curves [14,15].

**Figure 2:**
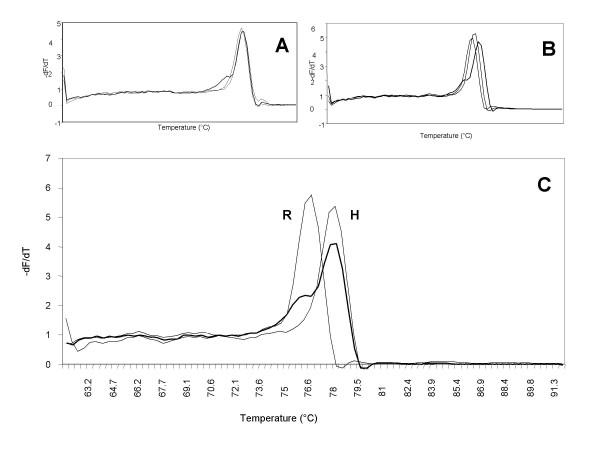
Tm analysis with SYBR^® ^Green I of 101-bp PCR products to discriminate the H/R alleles. Panel **A: **Melting profile without DMSO. Panel **B: **Melting profile with DMSO 5%. Panel **C: **Melting profile with DMSO 10%. Heterozygotes are depicted as thick lines, the heterozygous pattern is distinguishable from the two homozygous curves because of the shoulder, most likely due to the presence of heteroduplexes.

We tried to overcome those limitations and designed a pair of primers to amplify a 101-bp fragment including only the polymorphic positions in the coding sequence of *HA-1 *gene. Quality controls included the assessment of consistency in testing and re-testing the same specimens using independently generated PCR products. The Tm analysis with SYBR^® ^Green I was routinarily carried out in quadruplicate and turned out to allow discrimination of the H/R alleles by adding DMSO before the dissociation protocol (Figure 2, panel C). As depicted in panels A and B of Figure 2, the heterozygous pattern is clearly distinguishable from the two homozygous curves because of a shoulder, most likely due to the presence of heteroduplexes [12]. In our experience we obtained allele discrimination only by adding DMSO. It is well known that DMSO and other reagents such as formamide and glycerol can affect conformation and stability of nucleic acid strands. Indeed, we found useful to add DMSO in order to discriminate between H/R alleles and we believe that similar methods could find application in Tm analysis of certain PCR products where ins/del polymorphisms and/or SNPs are present.

## Conclusion

We detected mutations in novel sites relative to a consensus sequence of *HA-1 *locus. We showed that these variations are arranged onto few rare haplotypes possibly generated through recombination/gene conversion events from two major ancestral haplotypes. The observed sequence diversity, besides HA-1^R/H^, involves either a synonymous codon or intronic changes making them unlikely direct determinants of allogeneic recognition by cytotoxic T cells.

Probe-free, Tm assays most often require confirmation by other DNA mutation detection assays. However, we propose that Tm-based methods may be successfully applied to find experimental conditions, such as the use of DMSO described here, for genotyping ins/del and/or single nucleotide polymorphisms.

## Competing interests

The author(s) declare that they have no competing interests.

## Authors' contributions

C.G. carried out SSP-PCR allelotyping, performed sequence analyses and helped in drafting the manuscript. M.G. carried out the Tm assay, participated in the sequence alignment and helped in drafting the manuscript. C.M. participated in SSP-PCR allelotyping and in sequence analyses. P.L.M. participated in the design of the study and performed the statistical analysis. B.P. conceived the study, participated in its whole design and coordination and edited the manuscript. All authors read and approved the final manuscript.

## Pre-publication history

The pre-publication history for this paper can be accessed here:



## Figures and Tables

**Table 1:** HA-1 haplotypes found in 72 random chromosomes of the Italian population.

Name	N	c.427C/T	c.500C/T^§^	c.504A/G^§^	c.509+65G/A	c.509+130G/A	c.509+213 ins/del TTTAT
A^a^	39	C	T	G	G	G	ins
B^b^	27	C	C	A	G	G	del
C	4	C	T	G	A	A	ins
D	1	C	T	G	G	G	del
E	1	T	T	G	G	G	ins

^§ ^HA-1^R ^is defined by c.500T and c.504G; HA-1^H ^is defined by c.500C and c.504A (6)

^a ^This haplotype corresponds to the sequence reported under GenBank accession number AF092537

^b ^This haplotype corresponds to the sequence reported under GenBank accession number AF236756
